# Primary Angiitis of the Central Nervous System: New Potential Imaging Techniques and Biomarkers in Blood and Cerebrospinal Fluid

**DOI:** 10.3389/fneur.2019.00568

**Published:** 2019-06-06

**Authors:** Milani Deb-Chatterji, Simon Schuster, Vivien Haeussler, Christian Gerloff, Götz Thomalla, Tim Magnus

**Affiliations:** Department of Neurology, University Medical Center Hamburg-Eppendorf (UKE), Hamburg, Germany

**Keywords:** PACNS, biomarker, vasculitis, cerebrospinal fluid, peripheral venous blood, imaging, circulating endothelial cells

## Abstract

Primary angiitis of the central nervous system (PACNS) is an inflammatory brain disease affecting the medium and small vessels of the CNS. Although recent data of patients with PACNS have advanced the understanding of the disease, the diagnosis remains challenging. Clinical presentation of PACNS is broad and unspecific and the majority of the diagnostic approaches are hallmarked by a low specificity. Thus, PACNS is commonly misdiagnosed. In addition, due to its potential aggressive course which may be altered by an adequate immunosuppressive treatment, delineation from other vasculopathies and PACNS mimics is crucial. New diagnostic tools and biomarkers which increase specificity and facilitate the diagnosis for patients with suspected PACNS are highly desirable. This short review summarizes the current procedures within the diagnostic process and aims to illustrate its difficulties and challenges. Furthermore, it highlights emerging biomarkers in the cerebrospinal fluid and peripheral venous blood as well as novel potential imaging tools that may corroborate the diagnosis. With new imaging techniques and a panel of biomarkers the certainty of the diagnosis may be increased and diagnostic processes more accelerated in the future.

## Introduction

Primary angiitis of the central nervous system (PACNS) is a poorly understood disease which is restricted to the small- and medium-sized vessels of the brain and spinal cord. It is a rare disease with an estimated incidence of 2.4 cases per 100,000 person-years and an equal frequency in women and men (1). All age groups may be affected, whereas 50% are 37–59 years of age. Of note, PACNS is also a rare cause of stroke and remains an important differential diagnosis in younger (<45 years) adults (2.2%) (2, 3). Diagnosis is extremely hampered by the paucity of specific clinical symptoms and by the low specificity of the diagnostic approaches. Thus, establishing a reliable diagnosis remains challenging and PACNS is often misdiagnosed. In addition, two different subtypes of the disease have been reported, the small (SVV) and medium vessel variant (MVV), with different diagnostic phenotypes which increases the diagnostic challenge. While the MVV shows remarkable vascular irregularities in angiography procedures, current angiography techniques do not have a sufficient resolution to detect vessel abnormalities if the disease is limited to a small-vessel involvement. Moreover, since aggressive immunosuppressive therapy is mandatory to avert the often fatal course of the disease, exclusion from other etiologies is crucial. The aim of this short review is to give an overview of the current diagnostic procedures in PACNS and emphasize its difficulties. It further highlights new potential biomarkers in the cerebrospinal fluid (CSF) and peripheral venous blood as well as imaging markers that may increase the diagnostic accuracy.

## Current Diagnostic Approaches

The clinical presentation of PACNS is broad and unspecific. Thus, the average time to diagnosis may be several months (4). The symptoms vary from cognitive dysfunction, subacute, and progressive headache, seizures to focal neurological symptoms (5). Amongst others, the differential diagnosis includes e.g., hemorrhagic or ischemic stroke, neoplasia, encephalitis, dementia, migraine, multiple sclerosis. One of the most important differential diagnosis is the reversible cerebral vasoconstriction syndrome (RCVS) which bears the closest resemblance to PACNS with similar abnormalities in angiography. Recent studies provided new insights in diagnosing and distinguishing RCVS from PACNS by taking into account the patients' variables on admission (clinical symptoms, initial brain imaging etc.) (6, 7). However, diagnosis and distinction from this non-inflammatory vasculopathy is still difficult. In 1988, Calabrese and Mallek (8) proposed diagnostic criteria for PACNS which include a history or presence of a neurological deficit unexplained by any other cause after a thorough examination, evidence of vasculitis either by histopathology or angiography with characteristic changes of vasculitis and exclusion of a systemic vasculitis or any other condition to which the angiographic changes can be secondary. Birnbaum et al. (9) modified these criteria and differentiated between a “probable” and “definite” diagnosis. While definite PACNS is defined by histopathological evidence, all other cases are categorized as suspected PACNS. These revised criteria aimed at preventing inadequate and uncontrolled application of immunosuppressive treatment. To date, the diagnostic workup encompasses extensive laboratory examinations including an immunological screening and CSF analysis, MRI, angiography, and histopathology. Most of the tests are done to rule out differential diagnosis, such as systemic vasculitis (autoimmune or infectious), non-inflammatory vasculopathies (e.g., RCVS), or malignant disorders (e.g., lymphoproliferative diseases). Most of the patients (80–90%) show a pleocytosis (about 10–20 cells/ml) and/or increased protein levels (about 120 mg/ml) (4). However, these abnormalities in CSF are unspecific and, of note, an initial normal CSF does not exclude the diagnosis (10). In addition, there is currently no evidence of differences in CSF findings between the two disease variants. For brain imaging in PACNS, MRI remains the modality of choice. Fluid attenuated inversion recovery (FLAIR)—imaging, T1- and T2-weighted imaging, diffusion-weighted imaging (DWI), susceptibility-weighted imaging (SWI), time–of-flight MR angiography (TOF) and contrast medium-enhanced imaging are required sequences. Most of the patients with PACNS (90–100%) show abnormal MRI findings (9, 11). Typical changes include diffuse white and gray matter changes, contrast medium enhanced parenchymal lesions, leptomeningeal enhancement (12), parenchymal hemorrhages as well as microbleeds (13, 14). Tumor-like mass lesions have also been reported (15). Even a generalized atrophy in the MRI scan can be a sign of PACNS (16). Furthermore, MR angiography (MRA) can be applied. While in patients with SVV brain vessels in MRA are commonly unremarkable, vessel abnormalities are likely to be detected in MVV. Typical irregularities comprise peripheral alternating stenosis/occlusions and dilatation as well as vessel wall thickening in different vascular territories. However, none of these findings are specific, but a normal MRI scan in combination with a normal CSF result rule out the diagnosis of PACNS (17, 18). The current gold standard for vessel imaging in PACNS is digital subtraction angiography (DSA). Its reported overall sensitivity varies between 40 and 90% (4, 19, 20). This high range is most likely explained by the different subtypes of the disease (21, 22). Whereas, DSA may reveal remarkable vascular irregularities in patients with a predominant MVV, it shows unconspicious vessels in SVV. Currently, DSA has only a resolution for vessels >500 μm in diameter (23), thus, the SVV is typically “angiography-negative.” In addition, despite having a higher sensitivity in distal vessels and the posterior circulation (24) compared to MRA, the DSA provides information on changes in vessel contours only and not on the underlying pathological process (25). In particular, distinguishing non-inflammatory vasculopathies (e.g., RCVS or artherosclerosis) remains a challenge. Hence, DSA also has a low specificity. To date, brain biopsy is the only eligible technique to establish a definite diagnosis of PACNS. An optimal biopsy should contain samples from the dura, leptomeninges, cortex and white matter (26). However, the sensitivity of brain biopsy is also low (54–83%) (20, 21, 27). This broad range may be explained by the different case series including SVV and MVV. Notably, about one fourth of all autopsy-documented cases of PACNS were false-negative (28). In particular, in patients with MVV histopathology is frequently false-negative, probably due to abnormalities of medium-sized vessels that are rather detectable by angiography but not covered by brain biopsy (22). The rate of positive results can be increased by targeting MRI positive lesions (18). Three histopathological patterns are known in PACNS so far: granulomatous (58%), lymphocytic (28%) and necrotizing (14%), but they often overlap. The amyloid-ß angiitis (ABRA) with ß-amyloid depositions in the brain vessel walls is considered to be a subset of the granulomatous pattern. To date, no clear difference in clinical symptoms, aggressiveness of the disease, or treatment response between these patterns has been reported.

Due to the variable clinical presentation of PACNS, the lack of specific diagnostics and its differences between SVV and MVV (diverse DSA/MRA results, diverse biopsy results if derived from the right frontal lobe) a definite diagnosis is still a challenge. Additional diagnostic approaches are required to increase certainty. Thus, in the following we focus on potential, novel diagnostic imaging techniques and biomarkers in the CSF and blood to diagnose PACNS.

## Emerging New Diagnostic Biomarkers

### Interleukin-17 in the Cerebrospinal Fluid

**Figure 1:**
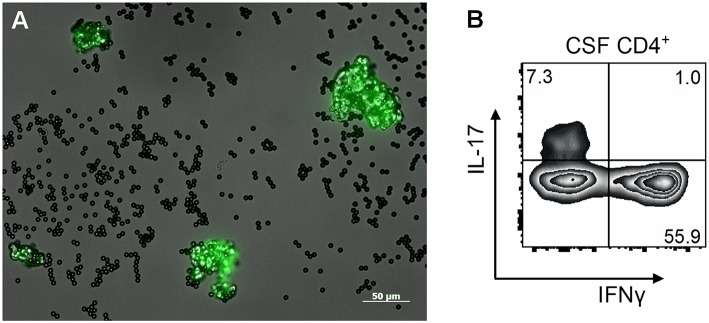
**(A)** Morphology of circulating endothelial cells (CEC, green) with Dynabeads™ (black) attached after immunomagnetic isolation from peripheral blood of a patient with PACNS displayed under a fluorescence microscope. CECs were isolated by CD 146-coated Dynabeads™ and with a fluorescence-coated (FITC) lectin (*Ulex europaeus agglutinin 1*, UEA-1) in an endothelial cell specific double-staining method. Afterwards CECs were enumerated in a counting chamber using a fluorescence microscope. **(B)** FACS plot of expanded T helper cells from a patient with biopsy proven PACNS. CSF cells were expanded in the presence of feeder cells, IL-2 und PHA for 2 weeks and an intracellular cytokine staining for IL-17 and IFNγ was performed.

A promising marker for PACNS in the CSF is interleukin-17 (IL-17). IL-17 is a pro-inflammatory cytokine and a potent mediator in cellular immunity. It plays a pivotal role in the pathogenesis of systemic vasculitis, in particular, antineutrophil cytoplasmatic antibodies (ANCA)-associated vasculitis (AAV), or giant cell arteritis (29). Previously, we reported that IL-17 produced by CD4+-T-cells in the CSF was elevated in patients with PACNS (sensitivity 73%, specificity 100%) (Figure 1A) (30). Elevated IL-17 levels were persistent in patients with active PACNS and patients in remission, thus indicating IL-17 being a more specific biomarker of cerebral vasculitis than the cell count or protein elevation in the CSF and being crucial also in the pathogenesis of PACNS. These findings merit the attempt of making IL-17 a target for novel therapeutic interventions. Interestingly, humanized anti-IL-17 antibodies as a treatment approach were already shown to induce clinically relevant responses in patients with psoriasis and rheumatoid arthritis (31). However, the promising results of IL-17 analyses in PACNS need to be verified in larger patient cohorts.

### Amyloid-beta A4 Protein (APP) in the Cerebrospinal Fluid

Mass-spectrometry-based techniques allow the assessment of a significant fraction of the proteome in biofluids without a pre-selection of target proteins. Ruland et al. reported a significantly lower abundance of proteins in the CSF of patients with PACNS (32). In particular, reduced APP concentrations were detected, a protein indicative for nervous system damage or pathology. Based on these findings the authors speculate that this protein may serve as a surrogate marker of brain injury in cerebral vasculitis. Further studies will have to validate these findings.

### Circulating Endothelial Cells in the Peripheral Venous Blood

Circulating endothelial cells (CECs) are known as markers of endothelial damage (33, 34). Inflammatory or non-inflammatory endothelial damage or mechanical injury can lead to the detachment of endothelial cells from the vessel wall. Thereafter, CECs can easily be detected in the blood by immunomagnetic isolation (Figure 1B) or flow cytometry (35). Preliminary studies of AAV showed increased CEC levels in patients with an active disease (36) whereas the number of CECs decreased under successful immunosuppressive treatment. Likewise, lower numbers of CECs were detected in patients with non-inflammatory vascular diseases, such as stroke and myocardial infarction (34, 37, 38). Of note, similar results were observed in patients with PACNS. We demonstrated that CECs were significantly elevated in patients with active PACNS while being decreased under successful immunosuppressive medication, in healthy controls and patients with stroke and cerebrovascular risk factors (39). Hence, CECs may potentially contribute to the diagnosis in biopsy negative cases and monitor the success of immunosuppressive treatment. However, although being a promising biomarker, studies with larger patient numbers are required to verify these results.

### High-Resolution Magnetic Resonance Vessel Wall Imaging (HR-MRI)

**Figure 2:**
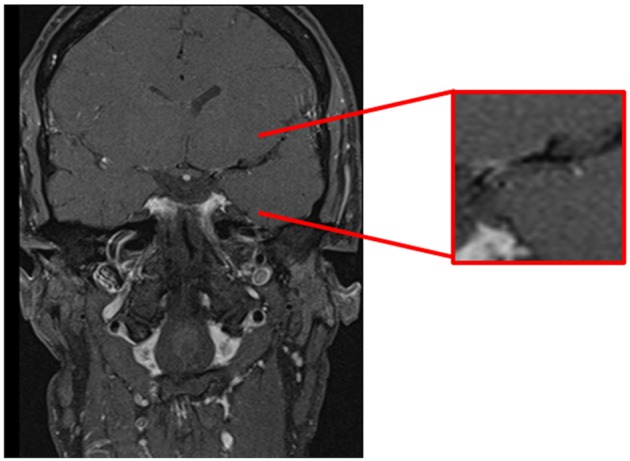
Cranial MRI, coronal T1-weighted-(dark-blood) sequence demonstrating contrast enhancement of the vessel wall in the first segment of the left middle cerebral artery.

Commonly used imaging modalities fail to distinguish between inflammatory and non-inflammatory vasculopathies. Preliminary studies demonstrated the possibility of outlining the vessel wall by contrast enhancement (40–42). In enhanced MR imaging of the vessel walls (“dark blood imaging”) the signal of the blood is suppressed and discrimination of the vessel wall from the lumen is increased (Figure 2). This HR-MRI can be performed as T1-weighted, T2-weighted or proton density-weighted sequences (43). This technique has already been used to distinguish PACNS from other vasculopathies (40, 42). In more detail, in PACNS a predominantly smooth, concentric, and long-segment wall thickening with strong enhancement and a perivascular edema were observed (44). Arteriosclerotic plaques exhibit a more eccentric, irregular, and short-segment wall thickening without perivascular edema and only mild enhancement depending on composition and activity of the plaque (25, 42). However, the distinction might still be difficult and this MRI sequence might rather help to differentiate PACNS from vasculopathies of young adults such as RCVS (41, 44). Notably, HR-MRI is merely capable of detecting the MVV with its current capacity of vessel resolution. However, this imaging technique is quickly developing and might soon lead to an improved discrimination between the MVV of PACNS and other intracranial vasculopathies (45). Of note, with higher resolutions it will be able to detect changes even in small vessels. Given that treatment can also affect the imaging findings, e.g., less enhancement under immunosuppressive or antiviral therapy (43, 46), it might also have the potential to monitor disease activity, which would make it extremely valuable for clinical trials (43, 47).

## Potential New Diagnostic Biomarkers

### Endothelial Progenitor Cells in the Peripheral Venous Blood

Another potential biomarker in PACNS are the endothelial progenitor cells (EPCs). In contrast to CECs, EPCs are considered to play a pivotal role in vascular regeneration and endothelial renewal (34). They are bone marrow-derived cells and originate from hematopoietic progenitor cells. EPCs are believed to be mobilized by various factors (e.g., vascular endothelial growth factor, VEGF), migrate to the site of endothelial injury, integrate in the endothelial cell layer and differentiate to mature endothelial cells (48). Interestingly, an inverse relationship of CECs and EPCs in patients with systemic vasculitis was previously reported (49). CEC values were highly elevated immediately after endothelial injury and decreased over time. In contrast, the EPC counts were low at time of endothelial injury and increased in the equal time course. Notably, similar results were observed in patients with Kawasaki disease, acute coronary syndrome and ischemic stroke (50–52). Hence, EPCs are presumed to be a marker for vascular or endothelial repair in inflammatory and non-inflammatory vasculopathies. However, EPCs have heretofore not been investigated, but these findings warrant the analysis of EPCs in PACNS.

### Von Willebrand Factor Antigen in the Peripheral Venous Blood

The von Willebrand factor antigen (vWF-Ag) is a plasma protein which is mainly synthesized from endothelial cells. Its function includes platelet aggregation and adhesion. If endothelium is damaged, e.g., by inflammation, increased levels of vWF-Ag may then be detected in blood. In fact, vWF-Ag was previously suggested as a potential biomarker in patients with systemic vasculitis. In patients suffering from Behçet disease and AAV, vWF-Ag levels were increased compared to controls (53–55). However, these elevated levels were observed in an active stage of disease and remained increased under remission. In contrast, in patients with Granulomatosis with Polyangiitis and Kawasaki disease vWF-Ag levels correlated well with disease activity (56, 57). Likewise, a recent study demonstrated that vWF-Ag may be a marker for disease activity in childhood PACNS (58). Elevated levels were detected in active disease and normalized under successful treatment. These results justify further analyses in adult PACNS.

### High Resolution Digital Subtraction Angiography

High resolution digital subtraction angiography including a 3D rotation technique provides a visualization of smaller vessels than with commonly used angiography techniques. A recent study demonstrated that this tool can display small branches (e.g., perforators) of the basilar artery (59). Fukuda et al. (60) described a fusion technique using two three-dimensional DSA images with precise information of the angio-architecture of arterial-venous malformations. The exact structure and location of the fistula, feeders, and drainers with high spatial resolution were obtained. Another novel technique to visualize small vessels beyond the spatial resolution of commonly used imaging modalities is the time-resolved 3D rotational angiography (4D DSA) (61). In particular, for proximal stenosis of the middle cerebral artery local collateral networks can be displayed and microangio-architecture in dural arteriovenous fistulas (dAVF) or arteriovenous malformations (AVM) can be visualized (62). Notably, 4D DSA might reduce the radiation and contrast agent dose and can be less time consuming compared to commonly used methods. In summary, novel imaging techniques in DSA could potentially provide more detailed information on smaller cerebral vessels due to higher spatial resolution. These imaging sequences were applied to studies of AVMs, dAVFs and aneurysms so far. They might also be applied to the SVV of PACNS to further increase the diagnostic specificity.

### Positron Emission Tomography (PET)

Fluor-desoxy-glucose (FDG)-PET is a functional imaging technique in nuclear medicine. It uses FDG as the radiopharmaceutical and depicts metabolic activity in the body by visualization of glucose uptake in tissues. Hence, inflamed vessel walls can be uncovered. In fact, in patients with biopsy proven giant cell arteriitis, FDG-PET was successfully applied and showed a high sensitivity and specificity (63). In addition, Novikov et al. demonstrated that PET can be a valuable diagnostic tool in patients with medium vessel involvement (64) and, thus, may contribute to depict inflammation of the vessel walls in patients with inconclusive diagnostic results (65). Of note, FDG-PET was previously applied to follow up and monitor disease activity in vasculitis (66, 67). However, small-vessel involvement cannot be detected by this imaging modality (68).

## Conclusion

This short review summarizes current diagnostic procedures applied to primary CNS vasculitis and aims at illustrating the difficulties and challenges in diagnosing PACNS. It further presents novel emerging and potential biomarkers which might facilitate the diagnostic process in the future. New biofluid and imaging markers may increase the certainty of the diagnosis. Biomarkers in the CSF (IL-17, APP) and peripheral venous blood (CEC, EPC, vWF-Ag) seem to be promising approaches, although potential differences in results between SVV and MVV remain to be determined. The ultimate goal would be to identify a panel of markers that might increase the diagnostic accuracy in PACNS in the future. In addition, novel potential imaging techniques, e.g., PET or different high resolution DSA-based techniques, can visualize smaller vessels or inflammatory vessel walls and, therefore, might support the diagnostic work. Data on these diagnostic tools are currently preliminary and, thus, need to be verified in larger patient cohorts or still need to be investigated in adult patients with PACNS. However, they might advance the diagnostic workflow in the future and aid to an accelerated diagnosis in patients lacking histological evidence, hence, in patients with suspected disease. They are promising diagnostic approaches that justify future attempts of investigation.

## Author Contributions

MD-C: substantial contribution to the conception and design of the work; acquisition, analysis and interpretation of data for the work; drafting the work and revising it critically for important intellectual content. SS: interpretation of data for the work; revising it critically for important intellectual content. VH: analysis and interpretation of data for the work; revising it critically for important intellectual content. CG: analysis and interpretation of data for the work; drafting of the work and revising it critically for important intellectual content. GT: analysis and interpretation of data for the work; drafting of the work and revising it critically for important intellectual content. TM: conception and design of the work; analysis and interpretation of data for the work; drafting of the work and revising it critically for important intellectual content. All authors provide approval for publication of the content. All authors agree to be accountable for all aspects of the work in ensuring that questions related to the accuracy or integrity of any part of the work are appropriately investigated and resolved.

### Conflict of Interest Statement

CG received fees as a consultant or lecture fees from Amgen, Bayer Vital, Bristol-Myers Squibb/Pfizer, Boehringer Ingelheim, Sanofi Aventis. GT received fees as a consultant or lecture fees from Acandis, Bayer Vital, Bristol-Myers Squibb/Pfizer, Boehringer Ingelheim, Daichii Sankyo, GlaxoSmithKline, and Stryker. TM received fees as a consultant or lecture fees from Boehringer Ingelheim, Novartis, Merck-Serono, Grifols, CSL-Behring and he received research fees from BMBF, DFG, EU, Werner-Otto-Stiftung, Schilling-Stiftung, Novartis, Merck-Serono. The remaining authors declare that the research was conducted in the absence of any commercial or financial relationships that could be construed as a potential conflict of interest.
